# Improved epitaxial growth and multiferroic properties of Bi_3_Fe_2_Mn_2_O_*x*_ using CeO_2_ re-seeding layers†

**DOI:** 10.1039/d3na00512g

**Published:** 2023-10-02

**Authors:** James P. Barnard, Jianan Shen, Yizhi Zhang, Juanjuan Lu, Jiawei Song, Aleem Siddiqui, Raktim Sarma, Haiyan Wang

**Affiliations:** a School of Materials Engineering, Purdue University West Lafayette IN 47907 USA hwang00@purdue.edu; b Sandia National Laboratories Albuquerque NM 87185 USA; c Center for Integrated Nanotechnologies, Sandia National Laboratories Albuquerque NM 87185 USA; d School of Electrical and Computer Engineering, Purdue University West Lafayette IN 47907 USA

## Abstract

In ferroelectric and multiferroic-based devices, it is often necessary to grow thicker films for enhanced properties. For certain phases that rely on substrate strain for growth, such thicker film growths beyond the typical thin film regime could be challenging. As an example, the Bi_3_Fe_2_Mn_2_O_*x*_ (BFMO) Aurivillius supercell (SC) phase possesses highly desirable multiferroic (*i.e.*, ferromagnetic and ferroelectric) properties and a unique layered structure but relies heavily on substrate strain. Beyond the thin film regime (approximately 100 nm), a less desirable pseudo-cubic (PC) phase is formed. In this work, a novel heterogeneous re-seeding method is applied to maintain the strained growth in this SC phase beyond the thin film regime, thus enabling the growth of thick BFMO SC phase films. The insertion of periodic CeO_2_ interlayers reintroduces the heteroepitaxial strain and effectively re-initiates the growth of the SC phase. The thick BFMO SC phase maintains the overall multiferroic and interesting anisotropic optical properties, even exceeding those of the typical 100 nm SC film. This re-seeding method can be effectively adopted with other SC systems or strain-dependent thin films, thus introducing practical applications of the new SC phases without thickness limitations.

## Abstract 2

The insertion of strain re-seeding CeO_2_ layers allows for thick growth of strain-dependent Aurivillius supercell phases.

## Graphical abstract

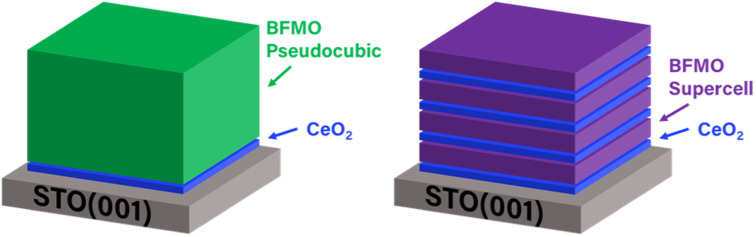

## Introduction

Multiferroic materials, those demonstrating two concurrent ferroic orders, *e.g.*, ferromagnetism and ferroelectricity, have been the subject of much research in recent years.^1–4^ Despite their potential uses in memories^5,6^ and spintronic devices,^7^ single phase multiferroic materials are scarce due to conflicting requirements of ferromagnetism (partially filled d-orbitals) and ferroelectricity (empty d-orbitals).^8^ Beside the well-studied single phase multiferroic materials, such as BiFeO_3_ (BFO)^9^ and BiMnO_3_ (BMO),^10^ a new group of Bismuth-based complex oxides, including Bi_3_Fe_2_Mn_2_O_*x*_ (BFMO),^11–18^ Bi_2_AlMnO_6_ (BAMO),^19–21^ Bi_2_MoO_6_,^22^ and Bi_2_NiMnO_6_ (BNMO),^23^ have shown interesting multifunctionalities, attracting attention and becoming the topic of many recent studies. Making this group of materials interesting is the growth of a unique anisotropic layered Aurivillius supercell (SC) phase and the resulting highly anisotropic properties.^13,17–20^ For example, BAMO has demonstrated anisotropic magnetic properties^19,20^ while BFMO has shown both anisotropic optical and magnetic properties.^13,17,18^

In many of this new group of SC multiferroic materials, substrate-introduced strain is considered necessary for the growth of their unique layered structures. For example, the BFMO system was previously examined *via* geometric phase analysis (GPA) to characterize the lattice strain variation across the film thickness, discovering that the growth of the BFMO on unbuffered LaAlO_3_ (LAO) substrate was accompanied by a highly strained pseudo-cubic (PC) BFMO interlayer before relaxing to the SC phase with ≈4% misfit strain.^12^ This strain can be introduced by the substrate, buffer layers, or a combination of both.^11,13,18^ This strain engineering method has been used to grow the BFMO SC film epitaxially on a range of substrates including silicon, a challenging substrate for oxide epitaxy.^11,18^ On the other hand, when the BFMO film is grown under low mismatch strain (*i.e.*, high epitaxial matching) conditions, the PC phase may be observed.^11,12,24–27^ The strained SC phases have been shown to possess more desirable multiferroic and anisotropic properties when compared with the PC phases.^16,17,26–28^

In general, strained film growth in epitaxial thin films is constrained to a critical thickness.^29^ Beyond this thickness, the interface-induced strain begins to relax and form misfit dislocations.^29^ This strain relaxation can lead to the growth of a new phase or crystal structure—starting at the relaxation point—that may not possess the desired properties. This thickness limitation becomes a roadblock if a greater film thickness is required, such as in ferroelectric applications, where larger total polarization and reduced leakage can be obtained with a thicker film. Magnetic and optical applications can also benefit from thicker films with stronger anisotropy and increased saturation magnetization. Optical anisotropy opens the door to applications such as polarizers, beam splitters, and modulators,^30^ while the enhanced magnetic saturation obtained by thicker films is used in certain micro-electro-mechanical system (MEMS) motor applications.^31^ Films must be capable of maintaining the desired properties over the needed thickness.

Aurivillius phases, such as the BFMO SC, have also been found to be particularly sensitive to strain and buffer layer structure.^12,13,18^ For example, if CeO_2_ is used as a buffer layer on top of the LAO substrate, the SC phase can grow immediately, due to the matching zigzagged bonding pattern between the CeO_2_ and the BFMO SC.^13^ This makes CeO_2_ an effective seeding layer for the BFMO SC, providing researchers with another dimension of freedom when tuning the film growth. Despite the success of SC layered oxide growth *via* substrate or buffer layer strain, most of the reported SC growths are thinner than 140 nm,^11,12,14,17^ while thicker films are desired for certain applications, including MEMS magnetic actuators,^31^ optical metasurfaces and photonic devices,^32^ and magnetoelectric antennas for energy harvesting.^33^ The questions remain: what factors limit the SC growth beyond 140 nm and how can we enable thick SC layer growth.

**Fig. 1:**
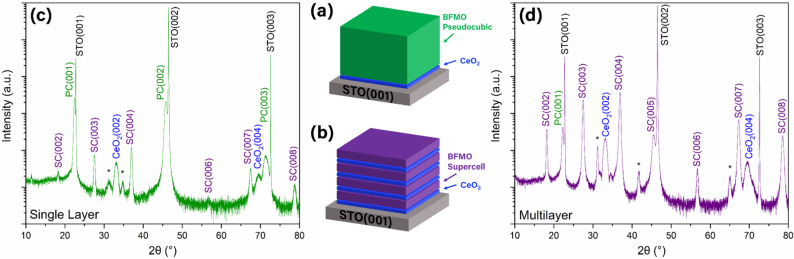
Film structure of (a and c) thick single layer and (b and d) multilayer films. (a and b) Schematic drawings and (c and d) XRD data are shown for each.

In this work, thick BFMO films (as thick as 275 nm, illustrated schematically in Fig. 1a) have been explored to understand the effects of strain on the SC growth and nucleation. A multilayer stack of repeating BFMO/CeO_2_ has been grown to test the idea of re-seeding the SC phase using CeO_2_ as the buffer layer, as illustrated schematically in Fig. 1b. As previously mentioned, the CeO_2_ buffer layer is known for its unique ability to seed bismuth-based SC layers due to the lattice similarities and mismatch strain. When the CeO_2_, which has a larger lattice (*a*_CeO_2__ = 5.411 Å), is grown on STO substrates (*a*_STO_ = 3.905 Å), a 45-degree rotation of the CeO_2_ has been observed due to the very close matching of the rotated CeO_2_ lattice (*a*_CeO_2__/2 = 3.826 Å).^18^ We theorize that a CeO_2_ interlayer can form a new nucleation surface for the growth of another BFMO SC layer. The BFMO SC layer is believed to grow at 45-degrees on top of CeO_2_ (no rotation relative to the substrate) due to a combination of domain and rotation matching epitaxy on the CeO_2_. The lattice parameters of the BFMO SC (*a*_BFMO_ = 7.98 Å in [100], *a*_BFMO_ = 11.97 Å in [010]) allow for 1 : 2 and 1 : 3 domain matching epitaxy, respectively, with the rotated CeO_2_ lattice (*a*_CeO_2__/2 × 2 = 7.652 Å, *a*_CeO_2__/2 × 3 = 11.478 Å).^11^ Schematic illustrations of the crystal lattice and atomic positions of the CeO_2_ crystal with the BFMO SC^14^ and PC^27,34^ phases are shown in Fig. S1,† including an enlarged image with the domain matching epitaxy between the rotated CeO_2_ lattice and the BFMO SC film. Additionally, Fig. S2† shows the unit cell epitaxy labeled with the lattice constants of each layer in the film stacks to demonstrate the strain that arises from the slight differences in lattice size. By inserting thin CeO_2_ layers periodically, the individual BFMO SC layers could remain thin while the total thickness of BFMO SC could exceed the current limit. The single layer and multilayer BFMO samples are compared for their phase purity, epitaxial quality, and ferroelectric and ferromagnetic properties. Such strain engineering based on this re-seeding process has not previously been applied to 2D layered Aurivillius phases, making this work important in harnessing the excellent multiferroic and optical properties of these new layered Aurivillius phases beyond the current thickness limit. This re-seeding method for growing thicker strained films could also be adopted for the growth of other well-studied SC materials and thus enable their practical applications.

## Experimental section

### Thin film growth

Pulsed laser deposition (PLD) was utilized to deposit the films of BFMO and CeO_2_ with a laser energy of 450 mJ, as measured at the laser source, and a background oxygen pressure of 50–200 mTorr. The films were grown on SrTiO_3_ (STO) (001) single-crystal substrates at temperatures of 650–850 °C. The BFMO target was constructed by pressing a pellet of Bi_2_O_3_, MnO_2_, and Fe_2_O_3_ powders and sintering at 750 °C for 3 hours. The PLD process was performed with a KrF excimer laser (*λ* = 248 nm) with an incidence angle of 45°. All films in a given sample were grown sequentially before removing the sample from the deposition chamber. A target-substrate distance of 4.5 cm was used in all depositions. Post deposition annealing was performed at 200 Torr O_2_ during sample cooling (10 °C min^−1^).

### Microstructure characterization

All films were initially characterized using X-ray Diffraction (XRD, PANalytical Empyrean) to determine their relative crystal quality. The surface roughness was measured using a Bruker Dimension Icon Atomic Force Microscope (AFM) with ScanAsyst-Air probes. Additional measurements were performed by Transmission Electron Microscopy (TEM) and High-Resolution Scanning TEM (HR-STEM) with Energy-Dispersive X-ray Spectroscopy (EDS) elemental mapping using the Thermo-Fisher TALOS F200X and the Thermo-Fisher Themis Z. The TEM samples were created using the grinding and dimpling method and ion milling polishing with the Gatan PIPS 695.

### Property characterization

The ferroelectric hysteresis loops were obtained using the Radiant Technologies Precision LC II Ferroelectric Tester. The 2D ferroelectric domain phase mapping characterizations were aquired through Piezoelectric Force Microscopy (PFM) using a Bruker Dimension Icon with SCM-PIT probes. The magnetic hysteresis loops were measured by the Quantum Design MPMS-3 SQUID magnetometer in vibrating sample magnetometry (VSM) mode. The ellipsometry measurements were performed using the J.A. Woollam RC2 spectroscopic ellipsometer. Various oscillators models were constructed to fit this data with the Mean Square Error (MSE) always below 5. Optical transmittance characterization was completed using the PerkinElmer LAMBDA 1050 spectrophotometer.

### Geometric Phase Analysis

The strain mapping analysis was performed using Gatan DigitalMicrograph (v1.83.842) with the GPA (v2.1) plug-in. HR-STEM images were used for the analysis. The power spectrum of the image was obtained and the diffraction spots corresponding to the in-plane and out-of-plane orientations of the reference material were selected. The STO was used as the strain reference for the single layer sample and for the first BFMO layer of the multilayer sample. For each of the stacked layers of the multilayer sample, the preceding BFMO layer was used as the reference, as its strain state was known from the initial STO referenced image. For the final GPA step, the strain field was calculated, resulting in the in-plane strain ε_xx_ images presented in this work.

## Results and discussion

A CeO_2_-buffered (10 nm CeO_2_) thick BFMO film (illustrated schematically in Fig. 1a) was grown on STO substrates by PLD under optimized conditions to a thickness of 275 nm, as measured by TEM. The CeO_2_ buffer has been used in previous works to seed the BFMO SC phase by providing the ideal strain conditions and a zigzag bond pattern like that of the BFMO SC.^13^ More specifically, the Ce–Ce bonds form a zigzag pattern that closely resembles the zigzag pattern of the Bi–Bi bonds in the BFMO SC phase.^13^ In this way, the CeO_2_ provides an ideal template for the BFMO SC phase. A 2*θ*–*ω* XRD scan was performed on this sample to better understand the crystal structure and determine if strain relaxation had occurred, shown in Fig. 1c. While several small SC phase peaks were identified, the highest intensity film peaks belonged to the PC phase of BFMO, which is believed to be the low-strain relaxed phase. This direct thick layer deposition confirmed that the BFMO SC phase relaxes to the PC phase at a thickness around 140 nm and beyond. It is interesting to note that very little BFMO SC phase remains in the sample. Based on the general mechanism for strain relaxation, the part of the film grown before the critical thickness should be in the SC phase, while the remainder should be in the PC phase. This will be discussed further in the TEM section below.

In contrast to the thick BFMO PC sample, the XRD data from the BFMO/CeO_2_ multilayer stack sample, given in Fig. 1d, shows high intensity BFMO SC peaks, but low intensity PC peaks, suggesting the successful growth of BFMO SC in the multilayer stack *via* CeO_2_ re-seeding layers. The CeO_2_ peaks had greater relative intensity as expected due to the larger total thickness of CeO_2_ in the sample. The peak locations also indicate highly textured growth of the CeO_2_, demonstrating that the growth on top of the BFMO SC layers was successful. An XRD rocking curve was also obtained for each sample using the SC(004) peak, shown in Fig. S3.† The full width at half-max (FWHM) for the multilayer sample was 0.700°, suggesting a relatively high epitaxial quality for such a thick film. The rocking curve for the single layer sample is also provided in Fig. S3† with a FWHM of 0.563°. Although this result appears unintuitive due to the superior quality of the SC phase multilayer sample, it is important to note that this rocking curve only captures information about the SC portion of the film. In the single layer film, there are very few small grains of the SC phase, which are close to the substrate. This leads to high orientation alignment and quality of the SC grains even though there is very little SC phase present. The lower intensity of the rocking curve data for the single layer sample confirms this.

**Fig. 2:**
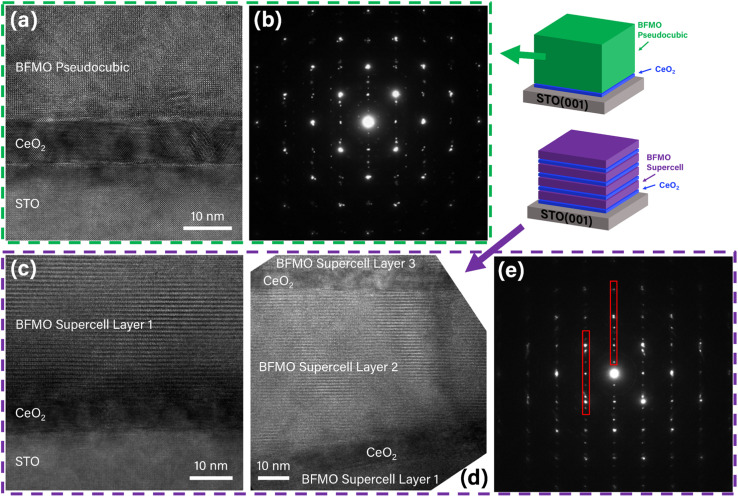
Electron microscopy images of (a and b) the thick single layer and (c–e) multilayer films. The thick single layer film (a) is identified as the pseudocubic phase while the first (c) and second (d) layers of the multilayer sample are identified as the supercell phase. The diffraction patterns of the single layer (b) and multilayer (e) indicate cubic and layered structures, respectively. The periodic our-of-plane diffraction spots marked in red in the multilayer sample (e) are typical of layered structures.

To better understand the microstructure and growth of the single layer and multilayer BFMO SC samples, TEM was used to image the samples. First, the thick single layer of the BFMO PC phase was studied, as shown in the cross-section TEM images in Fig. 2a and b. Consistent with the XRD results, the TEM images show that the thick film was grown in the less-desirable PC phase for the entire thickness. The selected area electron diffraction (SAED) pattern in Fig. 2b shows the typical diffraction spots that would be expected for the growth of a PC phase, with no indication of an anisotropic layered structure of the SC phase. TEM further shows the high epitaxial quality of the PC growth and demonstrates the important fact that the PC growth begins immediately at the CeO_2_ interface, rather than after some critical thickness. Small grains of the SC phase were observed scattered across the CeO_2_ interface, accounting for the small intensity SC peaks seen in the XRD data. Additional images of the film showing small SC grains and the PC phase through the entire thickness are shown in Fig. S4.† These findings suggest that the thick BFMO growth results in the PC phase becoming the most stable phase option. During the deposition, the stable PC phase formation in the higher region of the film can trigger a phase transformation of the already-deposited lower region of the film from the SC phase to PC phase. This SC-to-PC relaxation process could happen during the post-deposition annealing step around 650–750 °C. A future work incorporating an *in situ* measurement such as reflection high-energy electron diffraction (RHEED) could be very helpful to determine the timing of the change between the SC and PC phases.

The TEM images in Fig. 2c–e confirm the expected SC phase growth in the multilayer sample. The SC phase was observed with high epitaxial quality on the initial CeO_2_ buffer layer. The SC phase was again observed on top of the second CeO_2_ buffer layer, confirming the hypothesis of the capability of CeO_2_ to reestablish the required strain conditions. Additionally, the SAED pattern in Fig. 2e indicated a highly anisotropic layered structure with the appearance of satellite-like diffraction spots in the out-of-plane direction (indicated in red). Additional TEM images are provided in Fig. S5† showing the SC phase to be present in the remaining two layers as well. Energy-dispersive X-ray spectroscopy (EDS) was also used to confirm the chemical composition of the layers in both the single layer and multilayer samples, provided in Fig. S6 and S7,† respectively. The insertion of the CeO_2_ layers may also have an impact on the surface roughness of the film. To determine if this was the case, Atomic Force Microscopy (AFM) was used to measure the surface topography. The AFM area scan data is shown in Fig. S8† for each sample. The Root Mean Squared (RMS) surface roughness values for the single layer and multilayer samples were calculated to be 9.99 nm and 5.88 nm, respectively. The smaller surface roughness in the multilayer sample is likely due to the insertion of multiple CeO_2_ buffer layers. The CeO_2_ is known to grow in a planar fashion with a very smooth surface.^35^ The growth of the BFMO SC phase is more favorable on smoother surfaces, which is one component of the mechanism of the SC phase growth in this study.^13^

**Fig. 3:**
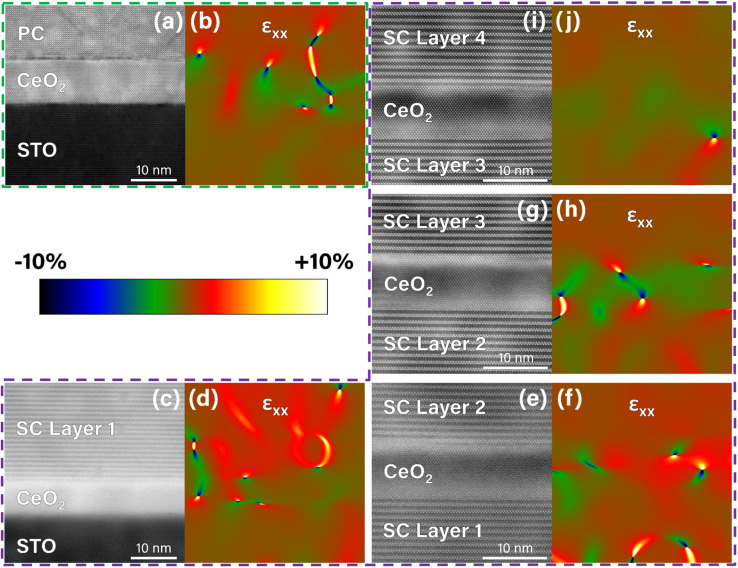
Geometric phase analysis strain images of (a and b) the thick single layer and (c–j) multilayer films. The HR-STEM image of each area is shown with the corresponding in-plane strain image, ε_xx_, where the spatial dimensions and physical area are the same. A color scale bar representing the strain is provided for the strain map images, which all have identical strain scales of −10% to +10%.

To better understand the strain effects on the stabilization of the SC phases between the CeO_2_ re-seeding layers, GPA was conducted on the interface areas between the BFMO and CeO_2_ layers in the single-layer and multilayer samples. The HR-STEM image used to study the single layer sample is shown in Fig. 3a, where the region of the film directly adjacent to the substrate and buffer layer is shown. The corresponding GPA in-plane strain ε_xx_ map is shown in Fig. 3b. For this analysis, the STO substrate was used as the reference material (defined to be zero strain). The mean strain in the BFMO PC region was calculated to be 0.687%. This value roughly matches the expected strain on STO substrates reported in a previous work on the BFMO PC phase.^11^ The relatively low strain (<1%) indicates excellent lattice-matched epitaxy, making the film stable at this greater thickness.^29^ This confirms that the PC phase is the relaxed phase of BFMO.

The GPA results across the first, second, third, and fourth CeO_2_ seed layers in the multilayer sample are shown in Fig. 3c–f, respectively. Beginning at the first layer of SC BFMO, shown in Fig. 3c, greater strain was observed than that of the PC BFMO case. Using STO as the zero-strain reference, the map, shown in Fig. 3d, indicates a mean strain of 1.301% in the BFMO SC phase. This epitaxial strain (between 1% and 7%) falls into the category of films with a relaxation after some critical thickness as expected.^29^ Since the actual strain is very close to the bottom end of this range, it would also be expected that the critical thickness would be substantial, again matching the several hundred nanometers that was observed. Since the STO substrate was not visible in the upper layer images in Fig. 3e–j and could not be used as the reference material, the lower layer of BFMO SC was used as the zero-strain reference for each image. This allows the relative strain through the total film thickness to be characterized. Since the BFMO SC phase is still present in each preceding layer, we assume that very little strain relaxation has occurred. The results show that the net mean strains of SC layers 2, 3, and 4 are 0.393%, 0.367%, and 0.299%, respectively. This indicates that some of the strain in each preceding layer has relaxed through its thickness, but that the entire original strain (≈1.3%) is reestablished at the CeO_2_ layer due to its re-seeding effect. These interesting results support the hypothesis of the strain stabilization on the growth of the BFMO SC phase through the CeO_2_ re-seeding process.

**Fig. 4:**
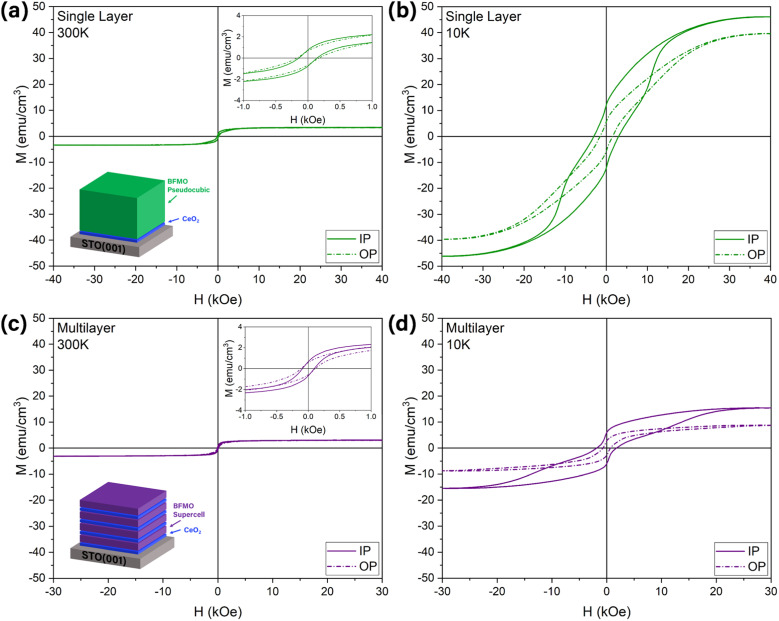
Magnetic property characterization of (a and b) the thick single layer and (c and d) multilayer films at (a and c) 300 K and (b and d) 10 K. In-plane and out-of-plane data is included for each sample and measurement condition.

The magnetic properties of the two samples were studied by measuring the magnetization in-plane (IP) and out-of-plane (OP). By sweeping the field over a broad range, a typical hysteresis loop can be obtained, shown in Fig. 4, providing the coercive field and remanent magnetization values. The magnetism in BFMO is believed to arise from the spin canting effect.^11,13,18^ The IP and OP magnetization values are similar; however, the IP direction tends to have superior coercivity and remanence. This easy-axis effect can be attributed to the anisotropic crystal structure in the SC phase. As expected, the difference is more evident in the multilayer sample since the majority of this sample is in the SC phase. The single layer sample appears to have less of a difference between the IP and OP directions since most of the sample is in the PC phase, which has an isotropic crystal structure. At 300 K, shown in Fig. 4a and c, the magnetic moments of each sample are similar. It is interesting to note that a significant difference is observed between the two samples at 10 K, shown in Fig. 4b and d. To better understand the reason, the effect of temperature on magnetization was further studied by a M(T) temperature scan, shown in Fig. S9.† The single layer and multilayer samples both show a relatively smooth curve as the temperature decreases. In the multilayer sample, it is likely that thermal perturbation is responsible for the entire increase as no sharp transition is present.^11^ However, in the single layer sample, a transition is observed around 150 K, where the magnetic moment begins to increase rapidly with decreasing temperature. This is likely due to the structure of the BFMO PC phase, where the body-center lattice site may be occupied by either an Fe atom or a Mn atom.^27,36,37^ When this lattice site inside a given unit cell is occupied by an Fe atom, the structure is essentially BFO for that unit cell. Conversely, if the site is occupied by a Mn atom, the structure resembles BMO. The BFO-resembling unit cells exhibit ferromagnetic Fe^4+^–O–Fe^4+^ interactions, which give rise to the transition near 150 K and explain the significantly higher saturation magnetic moment at 10 K.^26^ On the other hand, the BFMO SC phase does not possess these BFO interactions because the unit cell is completely different, therefore no significant low temperature transition is observed.^11^ Additionally, it has been previously reported that a thicker CeO_2_ buffer layer can have a negative impact on the magnetization of the BFMO SC.^13^ This is due to the increased surface roughness with thicker CeO_2_, which can cause the BFMO SC to become tilted, affecting the magnetic properties.^13^ The additional layers of CeO_2_ in the multilayer sample may be compounding this effect.

**Fig. 5:**
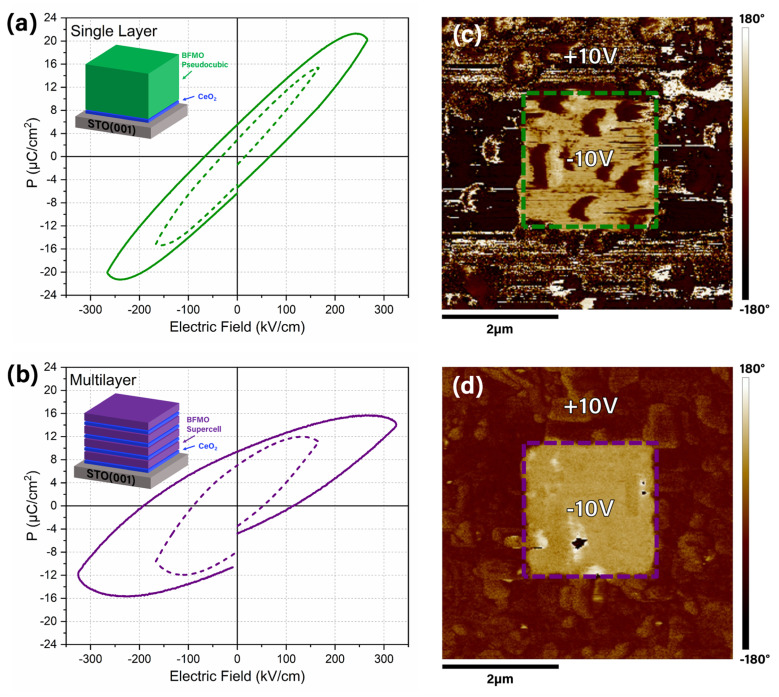
Ferroelectric property characterization by (a and b) *P*–*E* loops and (c and d) PFM area scans. The (a and c) single layer and (b and d) multilayer samples are characterized.

The ferroelectric properties of the BFMO films were characterized by two different methods. For the first measurement, a polarization–electric-field (*P*–*E*) loop was obtained *via* a standard ferroelectric tester. In this sequence, an electric field is applied to switch the polarization domains. In a good ferroelectric, hysteresis should be present with clear saturation.^38^ In leaky ferroelectrics, the loop will still have some hysteresis, but the saturation will be less obvious, with a characteristic “football shape”.^39^ The SC phase of BFMO is expected to have superior ferroelectric properties due to the Bi ions stacked in the Bi_2_O_3_ sheets of the Aurivillius phase, as reported in other works.^11,18,40,41^ First, the single layer sample, which is primarily in the PC phase, was measured. The hysteresis loop for this sample is shown in Fig. 5a. The lack of clear saturation indicates that the material is a leaky ferroelectric. The same measurement was performed on the multilayer sample, which is primarily in the SC phase, shown in Fig. 5b. The additional inserted CeO_2_ layers are not expected to have a significant impact on the ferroelectric properties as the sheet resistance of CeO_2_ is reported to be minimal when it is very thin.^35^ Studying the figures, the leakage is still present, but the coercive field and remanent polarization are larger than the single layer sample, indicating better ferroelectric properties. The slightly larger polarization in the single layer sample is attributed to the larger total thickness of BFMO in the single layer sample when compared to the multilayer sample, 277 nm and 240 nm respectively. The second method to analyze the ferroelectric properties is using PFM, where a DC bias on the tip is used to write the polarization state onto the sample surface followed by the application of a small, superimposed AC bias to probe the remnant polarization. If the oppositely orientated domains in two corresponding written areas retain their polarization orientation until the final read, the ferroelectricity of the sample can be identified. Starting with the single layer sample, the phase of the polarization is shown in Fig. 5c. While there is some contrast, indicating some remanent polarization, the contrast is low. This result agrees with the result from the *P*–*E* loop. The multilayer sample PFM scan is shown in Fig. 5d, where much more obvious contrast is visible, illustrating the well-defined polarized areas and again agreeing with the *P*–*E* loop data that the SC phase has superior ferroelectric properties. This measurement takes place on the minutes time scale, so leakage plays an important role. The BFMO SC phase has lower leakage than the PC phase due to the zig-zag atomic structure, resulting in superior phase contrast in the multilayer sample. This lower leakage can also be seen in the ferroelectric loop in Fig. 5b, where the remanent polarization at zero applied field is larger than in the PC phase. It is worth noting that these ferroelectric results still show a high degree of leakage, and the polarization falls behind cutting-edge ferroelectric such as HfO_2_.^42,43^ However, the quick switching of these films (low coercivity) could make them good candidates for low power device applications.

The optical properties were also measured to determine if these growth techniques have any impact on the overall optical properties. Ellipsometry measurements were performed for both samples, with the raw Psi and Delta data provided in Fig. S10.† The raw data was fitted using physical oscillator models and the resulting dielectric function for each sample is shown in Fig. S11.† Some anisotropic elements are visible, especially in the lower wavelength range (400–1000 nm), however the results of the two samples are very similar. The optical transmittance, which provides information about the bandgap of the materials as well as any plasmonic properties, is also provided in Fig. S12.† No clear absorption valleys or plasmonic resonances were observed. It is concluded that the SC and PC phases of BFMO have comparable optical responses.

The effectiveness of the CeO_2_ re-seeding approach for stabilization of the SC phase is clear when comparing the reported structural and functional properties of the single-layer and multilayer samples. The success of the CeO_2_ re-seeding layers could arise from multiple elements. The first element is crystallographic strain. The CeO_2_ re-seeding layer provides unique strain conditions and the zig-zag lattice pattern that favors the BFMO SC nucleation and growth. Second, the added CeO_2_ layers isolate the BFMO SC layers and maintained the thin SC layer structure of each sublayer. As previously discussed, the SC-to-PC phase transformations in the thick BFMO layer sample may occur during oxygen annealing and cooling. The insertion of CeO_2_ layers can halt this phase transformation as the individual BFMO layers are thinner and more stable.

This CeO_2_ re-seeding approach can also be used in other SC systems, such as the BAMO and BNMO Aurivillius phases, which have similar structures to the BFMO system.^21,23^ Another potential application for this technique is the combination of multiple Aurivillius phases using CeO_2_ interlayers. A preliminary demonstration is shown in the XRD data in Fig. S13,† where a BAMO film is grown epitaxially on top of a BFMO SC film using CeO_2_ to seed the new BAMO structure above the BFMO film. By enabling the epitaxial combination of multiple Aurivillius phases, desirable film properties from multiple materials can be harnessed simultaneously. The thicker multiferroic SC growth could allow practical applications of these new SC structures in devices where larger thicknesses are required such as MEMS magnetic actuators,^31^ optical metasurfaces and photonic devices,^32^ and magnetoelectric antennas for energy harvesting.^33^

## Conclusions

In summary, thin CeO_2_ re-seeding layers enable thick BFMO SC growth by utilizing the matching zig-zag lattice pattern between the two films. Compared to the single layer thick BFMO PC phase, the thick multilayer SC BFMO/CeO_2_ sample possesses enhanced magnetic and ferroelectric properties, as well as obvious optical anisotropy. This unusual method of phase stabilization *via* the re-seeding process may be tied to the unique properties of CeO_2_, namely heteroepitaxy with both STO and BFMO, strong oxygen ionic conductivity, and effective stabilization of the SC phase during the post-deposition annealing. The thicker BFMO SC film obtained by this innovative method will enable the practical applications of this film in magnetic field sensors, memories, actuators, and switches, which all require thick films for optimal physical properties.

## Conflicts of interest

There are no conflicts to declare.

## Supplementary Material

NA-005-D3NA00512G-s001
