# Supplementary material for: Improved epitaxial growth and multiferroic properties of Bi3Fe2Mn2Ox using CeO2 re-seeding layers
Source: Nanoscale Adv. 2023 Oct 2;5(21):5850–8. doi: 10.1039/d3na00512g (PMC10597562; doi:10.1039/d3na00512g)
Supplement: NA-005-D3NA00512G-s001 [file NA-005-D3NA00512G-s001.pdf]

Electronic Supplementary Material (ESI) for Nanoscale Advances.
This journal is © The Royal Society of Chemistry 2023

Improved Epitaxial Growth and Multiferroic Properties of $Bi_3Fe_2Mn_2O_x$ using CeO_2 Re-Seeding Layers

James P. Barnard,[1] Jianan Shen,[1] Yizhi Zhang,[1] Juanjuan Lu,[1] Jiawei Song,[1] Aleem Siddiqui,[2] Raktim Sarma,[2,3] Haiyan Wang[1,4,*]

[1]*School of Materials Engineering, Purdue University, West Lafayette, IN 47907, USA*
[2]*Sandia National Laboratories, Albuquerque, NM, 87185, USA*
[3]*Center for Integrated Nanotechnologies, Sandia National Laboratories, Albuquerque, NM, 87185, USA*
[4]*School of Electrical and Computer Engineering, Purdue University, West Lafayette, IN 47907, USA*
[*]*Corresponding Author E-mail: hwang00@purdue.edu*

Supporting Information:

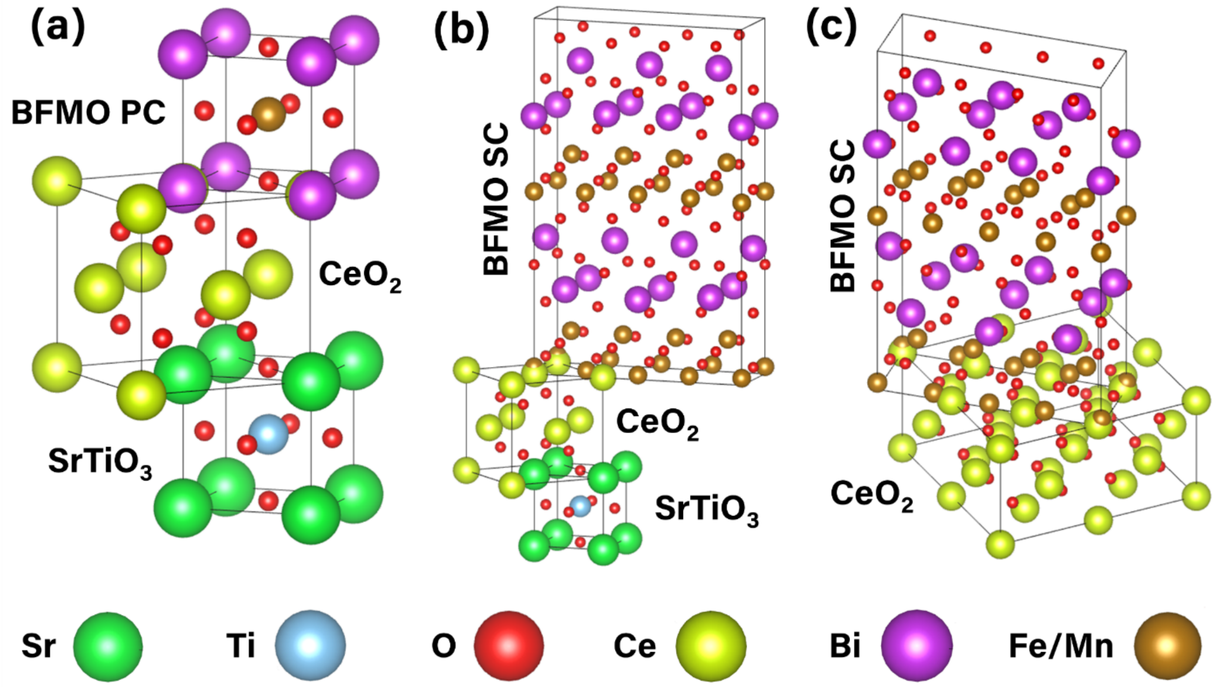

Figure S1. Crystal models of epitaxial matching between layers. (a) The matching present in the BFMO PC thick single layer film. (b) The matching present in the BFMO SC multilayer film. (c) An enlarged image showing the domain matching epitaxy between the rotated CeO_2 lattice and the BFMO SC lattice. The Fe and Mn atoms are randomly distributed through the Fe/Mn sites in both phases.

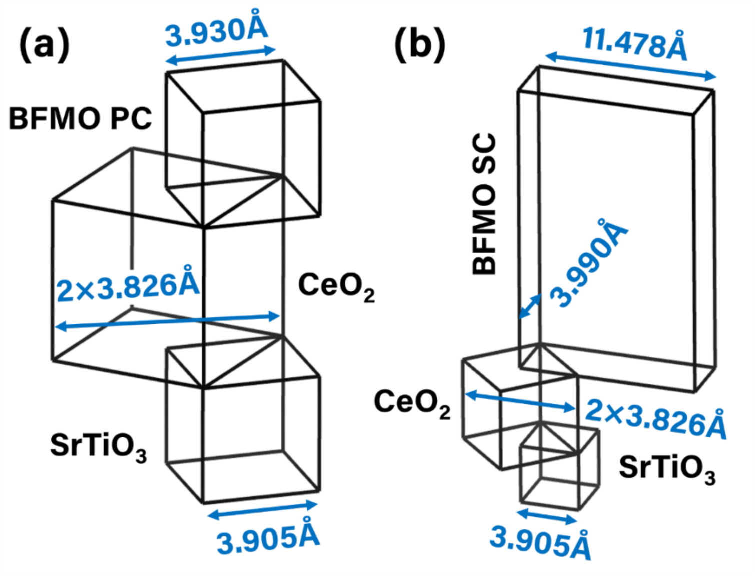

Figure S2. Unit cell models of epitaxial matching between layers. (a) The matching present in the BFMO PC thick single layer film. (b) The matching present in the BFMO SC multilayer film.

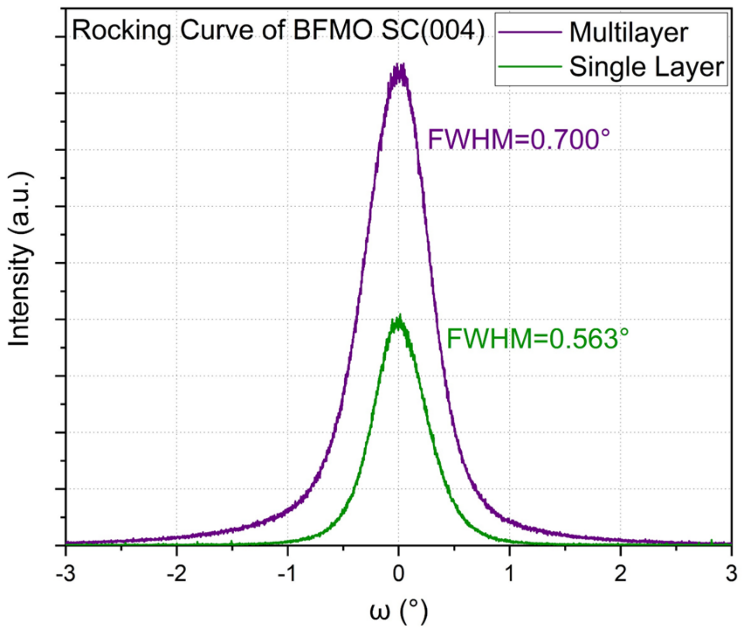

Figure S3. XRD rocking curves for single layer and multilayer samples.

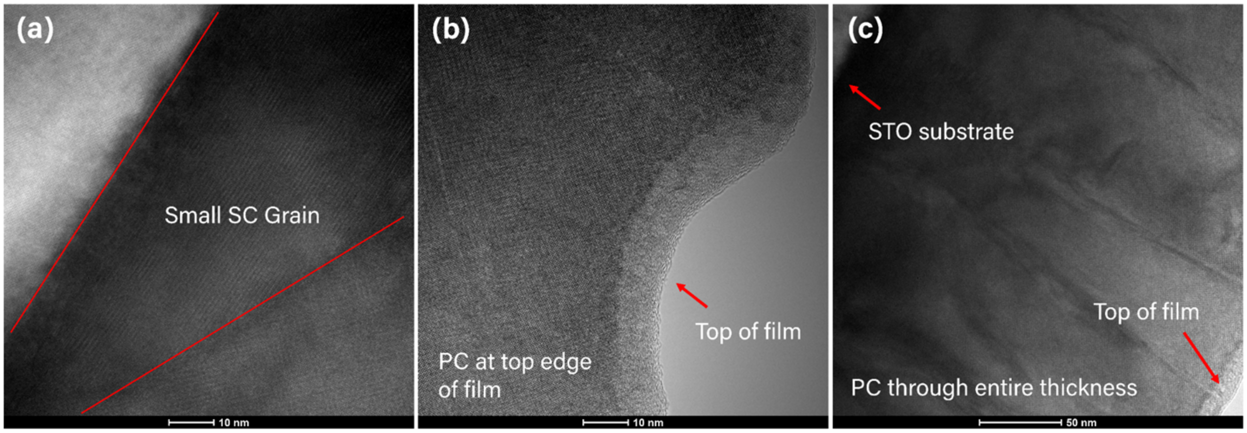

Figure S4. Additional TEM images of thick single layer sample showing large areas of the film at low magnification.

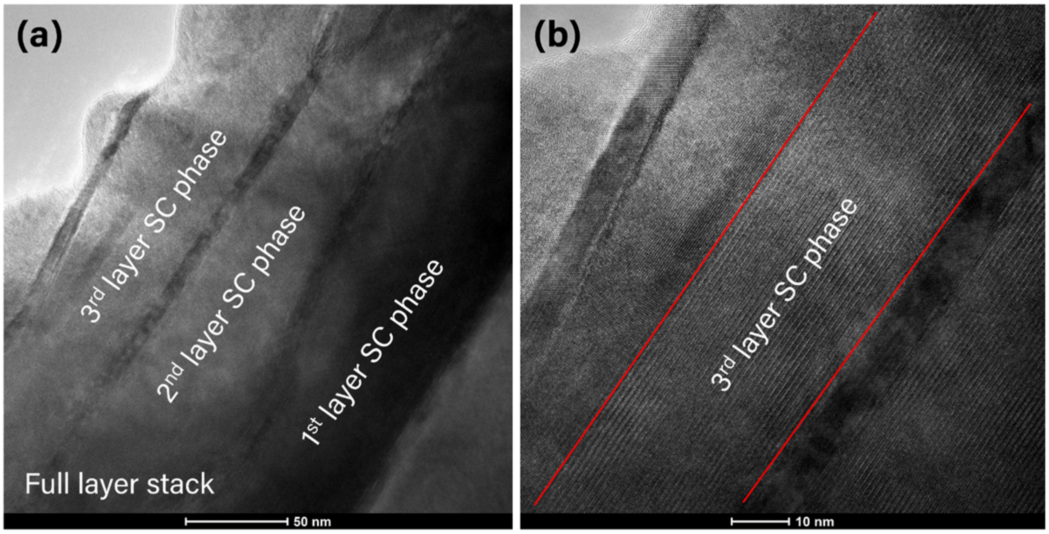

Figure S5. Additional TEM images of thick multilayer sample showing large areas of the film at low magnification.

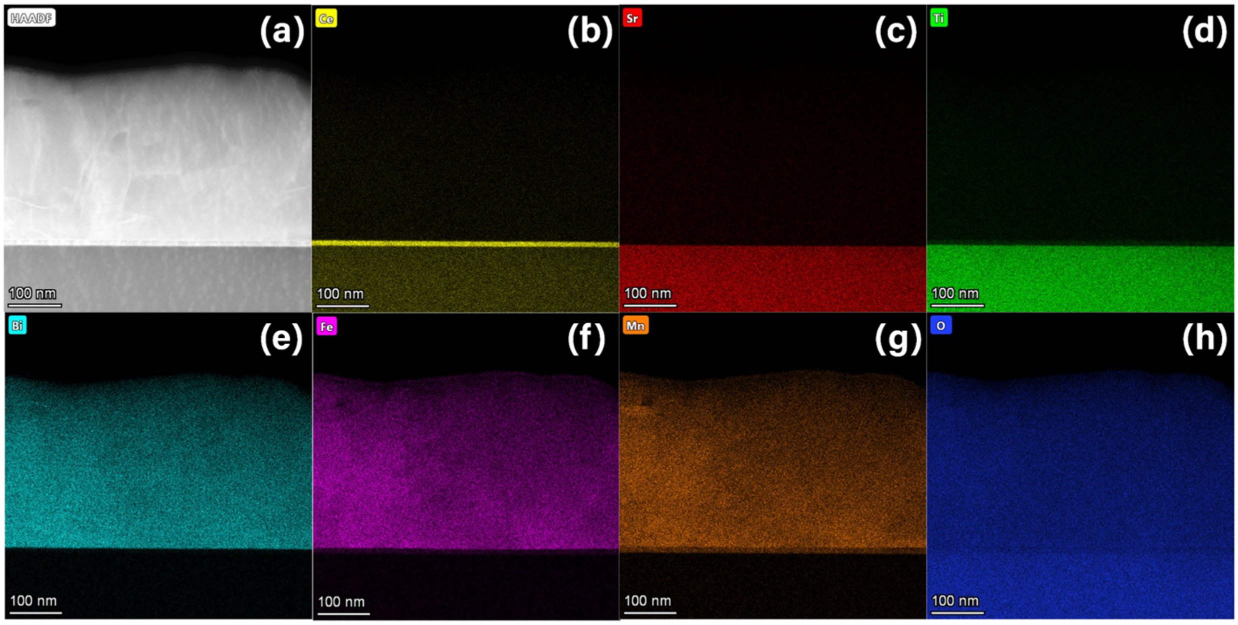

Figure S6. EDS mapping of thick single layer film using TEM.

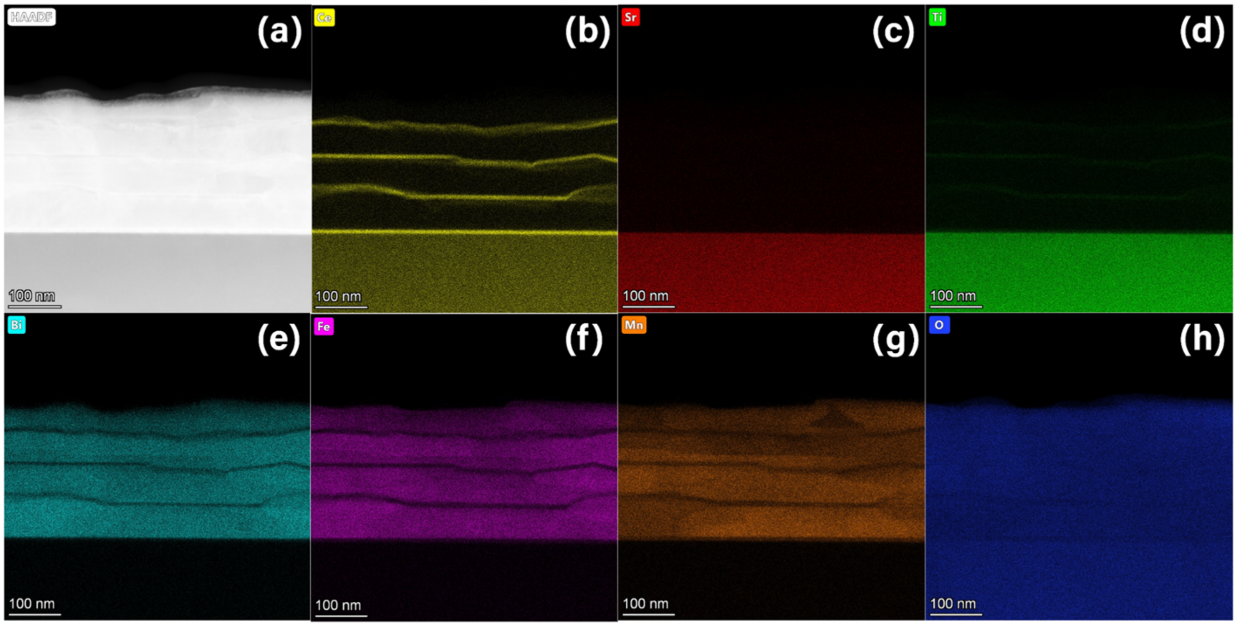

Figure S7. EDS mapping of thick multilayer film using TEM.

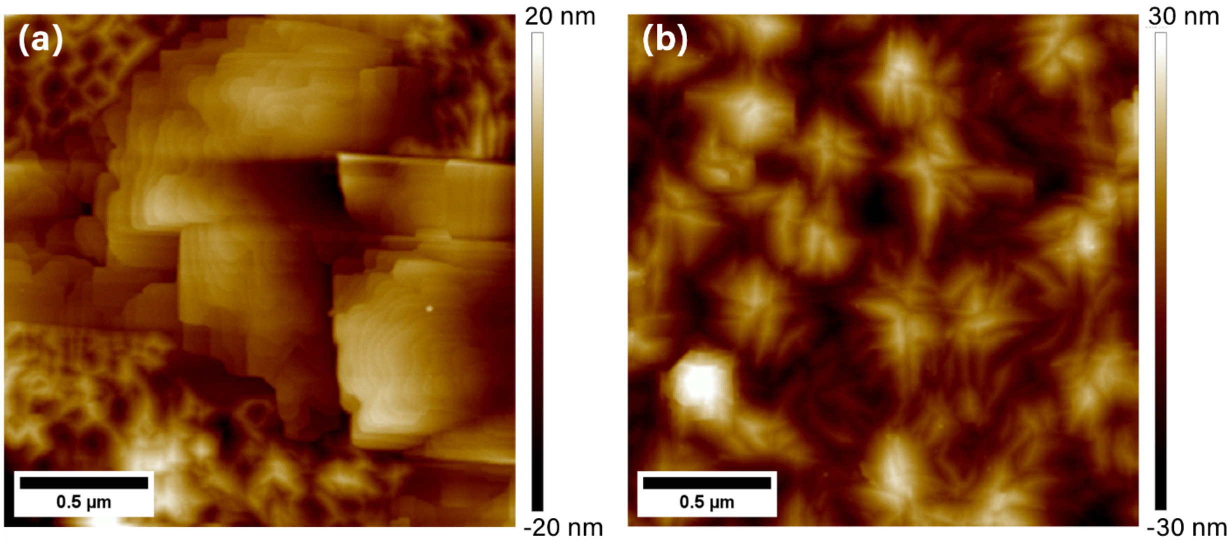

Figure S8. AFM surface topography maps of (a) multilayer and (b) single layer samples over a 2 µm x 2 µm area.

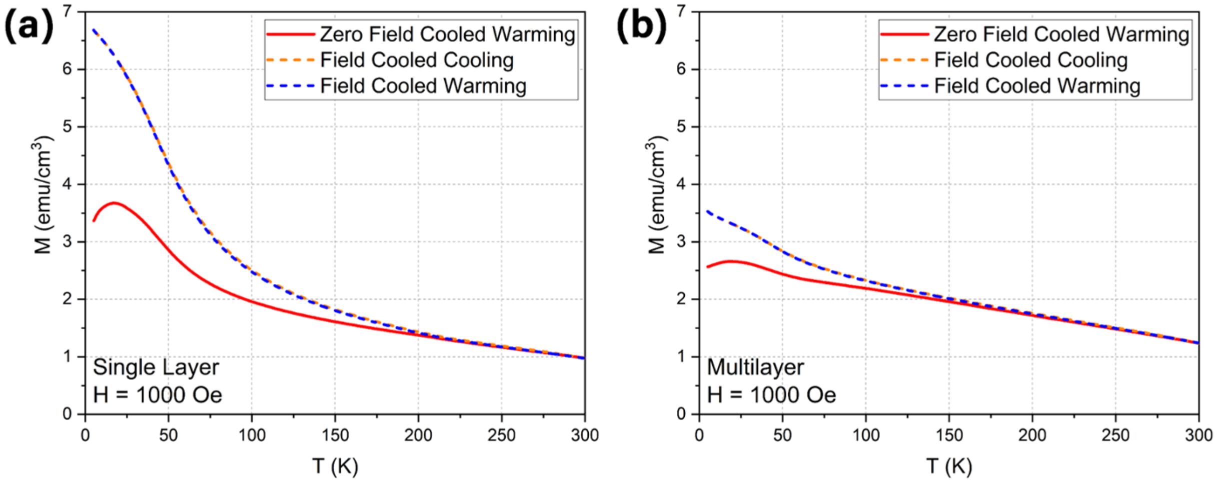

Figure S9. Magnetic temperature-dependent M(T) data for each sample including zero field cooled warming (ZFCW), field cooled cooling (FCC), and field cooled warming (FCW) curves.

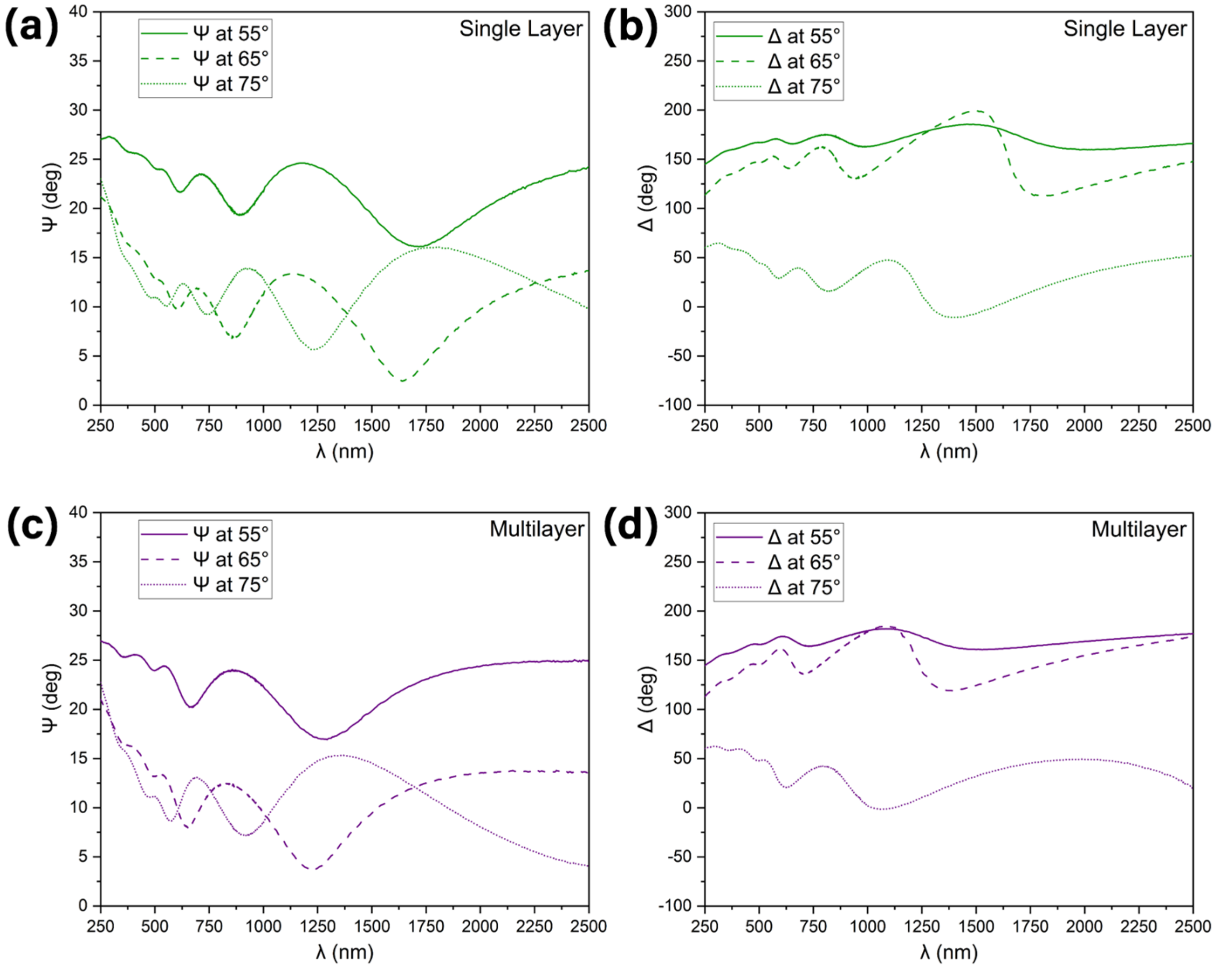

Figure S10. Raw ellipsometry optical data for each sample at various angles.

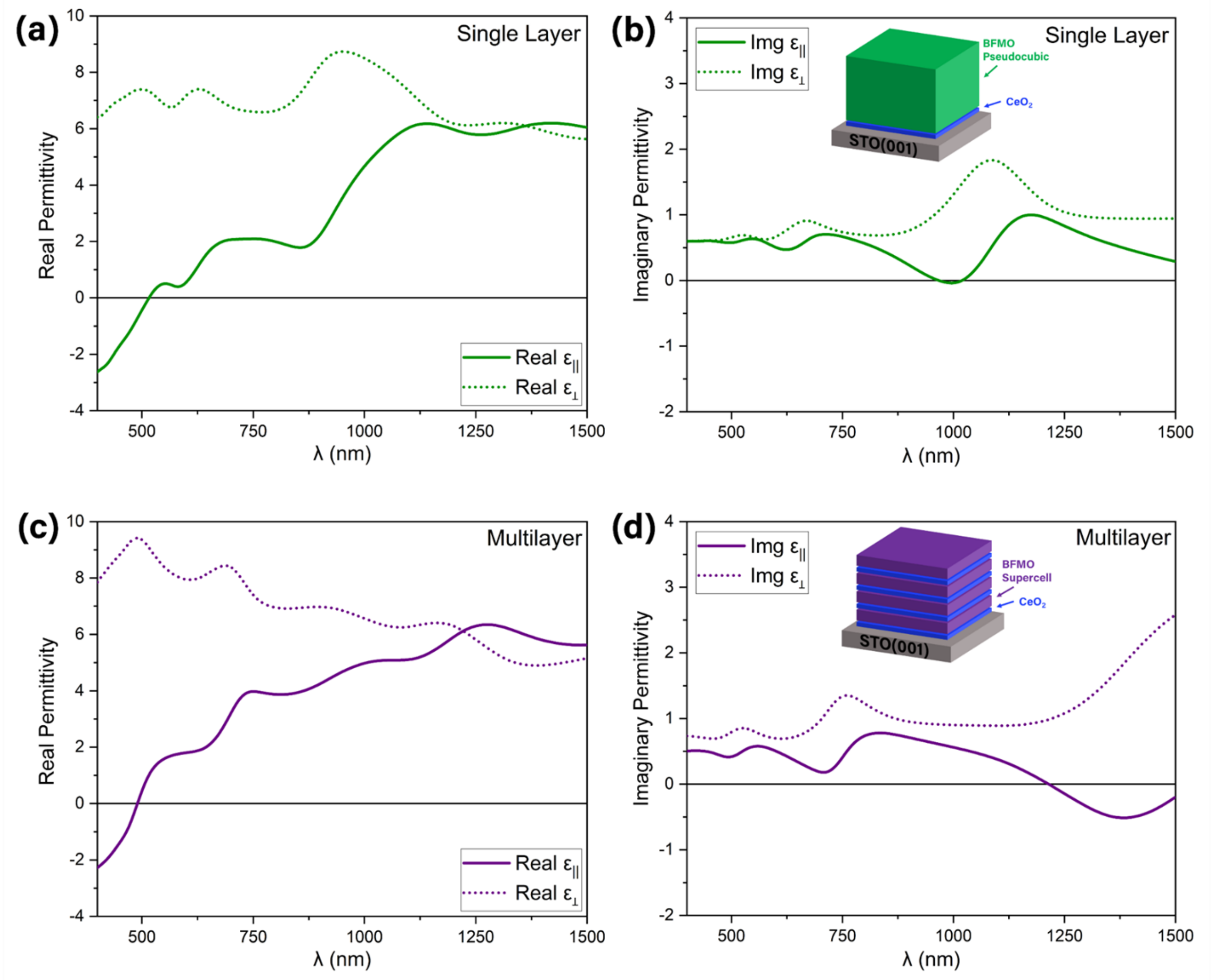

Figure S11. Modelled optical dielectric function of each sample including real and imaginary permittivity.

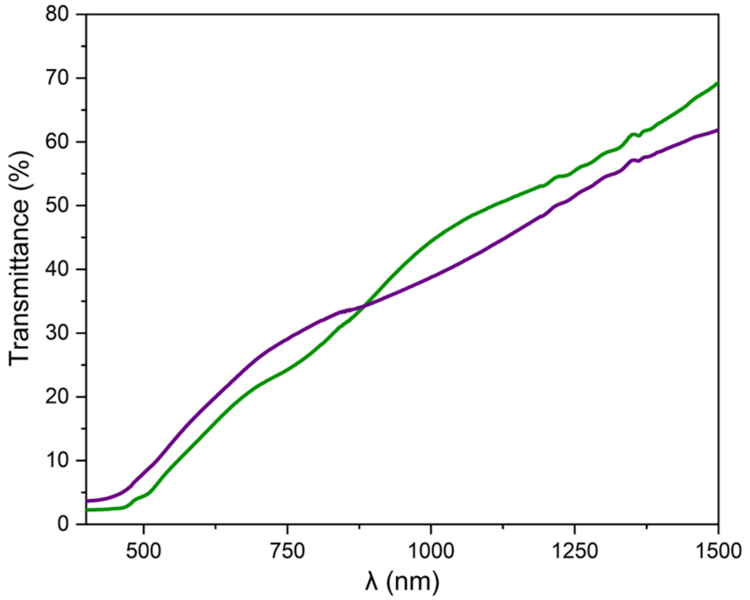

Figure S12. Optical transmittance data of single layer (in green) and multilayer (in purple).

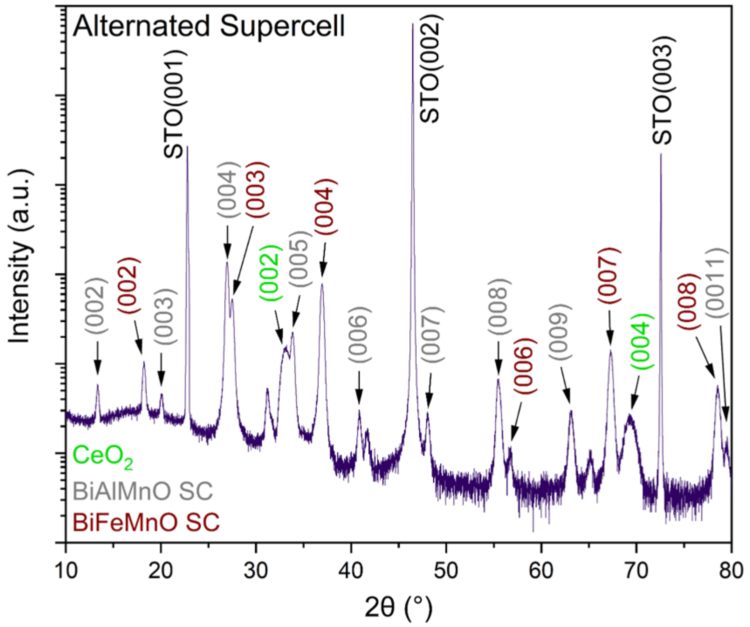

Figure S13. XRD data of alternating supercell structure with CeO_2 interlayers.
